# The Relationship Between Childhood Sexual Abuse and Executive Function for Those in Recovery from Opioid Use Disorder: A Brief Report of a Pilot Study

**DOI:** 10.1007/s10896-025-00876-3

**Published:** 2025-04-28

**Authors:** Laura Sinko, Paul Regier, Thais Costa Macedo de Arruda, Hasan Ayaz, Subash Aryal, Anna Rose Childress, Anne M. Teitelman

**Affiliations:** 1 Department of Nursing, Temple University College of Public Health, Philadelphia, USA; 2 Center for Studies on Addiction, Department of Psychiatry, Perelman School of Medicine, University of Pennsylvania, Philadelphia, USA; 3 School of Biomedical Engineering, Science and Health Systems, Drexel University, Philadelphia, USA; 4 School of Nursing, University of Pennsylvania, Philadelphia, USA; 5 Department of Psychology, College of Arts and Sciences, Drexel University, Philadelphia, USA; 6 Drexel Solutions Institute, Drexel University, Philadelphia, USA; 7 Center for Injury Research and Prevention, Children’s Hospital of Philadelphia, Philadelphia, PA, USA

**Keywords:** Childhood sexual abuse, Opioid use disorder, Executive functioning, Brain, Behavior

## Abstract

**Background:**

Both having an opioid-use disorder (OUD) and experiencing childhood sexual abuse (CSA) can significantly impact brain functioning, particularly within the executive system. Little research, however, has teased apart how individuals with compounding experiences may differ in brain functioning and behavior compared to those recovering from OUD alone.

**Aims:**

The purpose of this brief report was to understand the relationship between CSA and executive functioning in individuals with OUD.

**Methods:**

Participants were recruited from community organizations that serve those in recovery from OUD. Participants completed an in-person survey using an online platform (REDcap) and a series of cognitive tasks using the Penn Computerized Neurobehavioral Battery (CNB). Data from the survey and performance on the CNB were compared between those with and without a history of CSA.

**Results:**

Participants (*N* = 33) ranged in age from 31 to 60 years old (*M* = 45.56; *SD* = 8.67). Individuals with (CSA-Y, *n* = 20) vs. those without (CSA-N, *n* = 13) a history of CSA had significantly more mental health diagnoses (x2 = 7.98, *p* < 0.01) and depressive symptoms (t = 3.1, *p* < 0.01). The CSA-Y group had fewer correct responses than CSA-N for the Short Penn Line Orientation task (sPLOT, t = 2.5, *p* = 0.02). There were no other significant differences in the other CNB tasks. Adding depressive symptoms to the model made the CSA / sPLOT relationship non-significant. There was a tendency for depression symptoms to moderate the CSA / sPLOT relationship (change in R^2^ = 0.05, F(1,29) = 3.1, *p* = 0.09). The CSA-Y group (*R* = −0.57) had a significant inverse relationship between depressive symptoms and sPLOT correct responses, but the CSA-N group did not.

**Conclusion:**

Those with a history of CSA who are in recovery from OUD may have difficulties with executive functioning, particularly spatial reasoning compared to their recovering peers. The way depression manifests for these individuals is important to consider when designing interventions to support the mental and behavioral health of these populations.

## Introduction

Opioid use disorder (OUD) is a chronic condition characterized by the persistent use of opioids, including prescription pain medications (e.g., oxycodone, hydrocodone), synthetic opioids (e.g., fentanyl), and illicit drugs such as heroin. Symptoms of OUD include cravings, withdrawal, difficulty in controlling opioid use, and challenges in fulfilling personal and professional obligations (Amari et al., 2011). In 2020, over 2.7 million people in the United States reported having OUD (CDC, 2021), with numbers increasing significantly during and after the COVID-19 pandemic (NIDA, 2022; CDC, 2022). The primary risk associated with OUD is death by overdose, with approximately 75% of the over 100,000 overdose deaths in 2021 being opioid-related (CDC, 2022). The city of Philadelphia, in particular, has one of the highest rates of death by overdose within large cities in the US with over 38,000 residents exhibiting current or past OUD diagnosis (Philadelphia Department of Public Health, 2021).

Histories of trauma frequently co-occur in individuals with OUD, potentially increasing vulnerability to overdose and death. For example, individuals who have experienced trauma may use opioids as a coping mechanism to manage emotional pain and distress (McHugh et al., 2016; Austin & Short, 2020), contributing to the development of OUD. Studies have also revealed that those with sexual or physical trauma during childhood displayed greater risk for addiction, and the severity of the trauma had a significant correlation with depression and substance use disorder (He et al., 2022; Schäfer et al., 2000; Rosen et al., 2002). Furthermore, extensive literature showed that persons with OUD are at greater risk of developing mental health issues, and viceversa (Sullivan, 2018; Scherrer et al., 2016; Amari et al., 2011). Depression, in particular, has been shown to be both a risk factor for longer and more severe use of opioids (Sullivan, 2018), as well as a potential consequence of OUD, as the prevalence of this condition was found to increase over time in patients with OUD (Kosten & George, 2002).

### OUD and Childhood Sexual Abuse

Childhood sexual abuse (CSA) encompasses any sexual activity involving a child, ranging from inappropriate touching or exposure to penetrative acts, which the child cannot comprehend or consent to, with anunderestimated prevalence of 12% of children worldwide (Stoltenborgh et al., 2011). Childhood is a pivotal time in human development, with critical physical and neural changes occurring (Tierney & Nelson, 2009). Consequently, experiencing sexual violence during this period can be especially harmful, as it may lead to life-long consequences at the level of the brain (Wilson, 2010). The psychological effects of childhood abuse can evolve into lasting psychopathologies such as post-traumatic stress disorder, depression, eating disorders, substance use, impulsivity challenges, anxiety symptoms, and suicidal tendencies (Hymen et al., 2008; Miller et al., 1993; Wilson, 2010). Research has shown that CSA, especially when involving a family member, is associated with more negative health outcomes than other forms of abuse. Current research suggests that people who abuse opioids report high rates of adverse childhood experiences, including childhood sexual abuse, as well as sexual assault in adulthood and mental health problems such as post-traumatic stress disorder, suicidal thoughts, and psychological distress (Austin & Shanahan, 2018; Engstrom et al., 2012; Schiff et al., 2010). Studies have found that experiencing CSA not only increases the likelihood of developing mental health disorders (e.g. Mullen et al., 1993), but it also results in longer lasting symptoms of depression (Zlotnick et al., 2001).

The link between CSA and OUD is particularly strong and complex. Individuals with a history of CSA may be more prone to engaging in substance use to manage the emotional distress associated with those experiences (Wilson, 2010). This relationship may be uniquely potent compared to other forms of childhood trauma due to the intensely personal and boundary-violating nature of sexual abuse. CSA can lead to profound feelings of shame, self-blame, and loss of control (MacGinley et al., 2019), which may drive individuals to seek the numbing and dissociative effects of opioids specifically. Moreover, the neurobiological changes associated with CSA, including alterations in stress response systems and reward circuitry (Andersen et al., 2008), may increase vulnerability to opioid dependence. Importantly, studies suggest that CSA may have particularly strong associations with dissociative symptoms and alterations in reward processing, which could be especially relevant to opioid use and addiction (Teicher et al., 2010; Dannlowski et al., 2012). These neurobiological changes may contribute to the heightened risk of opioid dependence among CSA survivors.

Consistent with this idea, it is estimated that among persons with OUD, 41% of women and 16% of men have experienced CSA (Santo et al., 2021). These statistics may be vastly underestimated, however, as disclosure of CSA, particularly among men, is extremely low. Studies have found that opioid-dependent individuals are more likely than non-opioid-dependent individuals to report experiencing childhood sexual abuse, as well as other forms of abuse and neglect. Among those who reported experiencing sexual abuse, opioid-dependent respondents were more likely to indicate that they experienced penetrative sexual abuse and multiple incidents of sexual abuse (Conroy et al., 2009). Engstrom et al. (2012) found high rates of childhood sexual abuse (56.9%) among women in methadone treatment. The higher prevalence of CSA among women with OUD, compared to other forms of substance use disorders, suggests a unique relationship between these experiences (Conroy et al., 2009). For example, results from previous studies show an association of CSA with prescription opioid misuse (Guo et al., 2017; Quinn et al., 2016; Austin & Shannahan, 2018). This may be due to the particular effectiveness of opioids in managing the specific type of emotional and physical pain associated with CSA, as well as their ability to induce a state of detachment that may be especially appealing to CSA survivors (Khoury et al., 2010).

### CSA and OUD Impact on Cognition

Exposure to early life adversity is believed to impact cognition, including executive functioning, psychomotor, and visuospatial abilities (Kavanaugh et al., 2017). However, the specific cognitive outcomes and directionality associated with CSA remain unclear. Several studies revealed that individuals with a history of CSA perform more poorly in tasks of visuospatial and psychomotor abilities (Majer et al., 2010; Spann et al., 2012; Navalta et al., 2006). Another recent study by Letkiewicz et al. (2021) revealed a direct relationship between childhood abuse and executive functioning, such that greater cumulative abuse was associated with poorer executive functioning. Yet, consensus is lacking as other researchers point out “normal” cognitive functioning in adults who experienced CSA (Feeney et al., 2013; Porter et al., 2005). In addition, the impact of disorders like depression that frequently co-occur with CSA and OUD are still being examined. For example, one study revealed individual impacts of prior adversity and depression on cognition but not a combined effect (Koopowitz et al., 2021). It is unclear whether depression will have an impact on the putative relationship between CSA and cognitive outcomes.

Alterations in brain connectivity following traumatic experiences are believed to be associated with challenges in cognitive functioning. From a neurological standpoint, research has shown that childhood trauma is directly associated with changes in hippocampal plasticity (Bremner et al., 1997; Brunson et al., 2005), which is implicated in the learning process (Preston et al., 2004) as well as in spatial memory and spatial orientation (Smith & Milner, 1981). Hence, it is reasonable to expect that CSA could result in later challenges in those same areas.

The impact CSA has on cognition may be compounded when individuals also experience OUD. According to the Cognitive Deficits Model of substance use, individuals with substance use disorders can exhibit alterations in the brain that result in cognitive impairments related to executive functioning (Wollman et al., 2017). In line with this, reviews of existing literature have demonstrated that persons with OUD display significant impairment in areas of spatial and psychomotor abilities, as well as cognitive flexibility. For example, a study conducted by Tolomeo et al. (2019) showed that patients with current OUD displayed significantly inferior visuospatial memory performance as compared to persons without OUD (abstinent and control groups). In another study, chronic opioid users displayed considerably poorer pattern and spatial recognition compared to control subjects (Ornstein, 2000). In addition, a recent meta-analysis revealed that patients with OUD exhibited significant impairments in psychomotor abilities, with this domain showing the largest effect size difference among the various abilities that were evaluated (Wollman et al., 2019). Relatedly, cognitive flexibility, defined as the ability of switching between different tasks or mental spaces in response to setting changes or new information, has been shown to be impaired in patients with OUD, particularly as it relates to attention shifting (Ornstein, 2000; Hekmat et al., 2011). Yet, it remains unclear how these cognitive effects of both childhood trauma and OUD may compound and interact with one another.

### The Current Brief Report

Although multiple lines of research have investigated the impact of OUD on cognition, there are few studies that consider the co-occurrence of OUD and childhood trauma to understand that relationship. Moreover, looking at certain abuse types specifically (e.g., CSA) takes into consideration that different types of traumas may evoke different outcomes. Hence, it is vital to further understand the interplay of all of these factors, in addition to other co-occurring consequences (e.g., depression). This brief report aims to analyze the relationship between CSA and executive functioning in a population with OUD. It also aims to understand how depression might manifest in individuals with co-occurring CSA and OUD, and how these symptoms impact these relationships. We hypothesize that individuals with a history of CSA will display more challenges in completing executive functioning tasks compared to their counterparts without a history of CSA, and that their depression symptom burden will significantly impact that relationship. This hypothesis is aligned not only with previous literature, but also with a pilot study conducted within our laboratory that presented sustaining evidence for this further analysis (Regier et al., 2022).

## Methods

We used an online survey and cognitive battery to understand the relationship between CSA and executive functioning. Individuals (*n* = 33) were recruited via flyers, email listservs, word of mouth, and presentations at a local methadone clinic and in-person programmatic events. Those who were interested reached out by phone or email to be screened for our study. Inclusion criteria for this study were: (1) ages of 18–60, (2) on MAT for OUD, and (3) using opioids in the past year. Exclusion criteria were: (1) being currently pregnant, (2) being not stable on mental health medications, and (3) currently being in a manic state. Prior to participation, written informed consent was obtained from all subjects, and all procedures in this study were approved by The University of Pennsylvania Institutional Review Board. The online survey and Computerized Neurocognitive Battery (see section on CNB below) were completed at a local community organization serving those in recovery from OUD. This session lasted approximately 2 h and participants were compensated for their time.

### Survey Instruments

*Demographic and health variables* measures included age, zip code, gender, race, ethnicity; socio-economic measures included mother’s highest level of education, current employment, government assistance, and income. Drug use was assessed by asking whether individuals had used alcohol, cannabis, opioids, and/or stimulants in the past year and, if so, for how many years.

*CSA* was measured using an expanded version of the Adverse Childhood Experiences (ACEs) survey (Cronholm et al., 2015). The questions used to assess sexual abuse during childhood were: “Before the age of 18, did an adult or older relative, family friend, or stranger who was at least five years older than yourself… Ever touch or fondle you in a sexual way or have you touch their body in a sexual way?” and “Attempt to have or actually have any type of sexual intercourse, oral, anal or vaginal with you?”. Participants who selected “yes” for either of those questions were considered to have a history of CSA (CSA-Y), and participants who selected “no” for both of those questions were considered to not have a history of CSA (CSA-N).

#### Mental health diagnosis history.

Participants were asked to self-report their mental health diagnosis (e.g., depression, anxiety, etc.) through the following inquiry: “Have you been diagnosed with a mental health disorder?“.

*Depression* was measured two ways: using the Quick Inventory of Depressive Symptomology (QIDS) (Rush et al., 2003) and the PROMIS Item Bank v. 1.0 - Emotional Distress–Depression (PROMIS-DEP; Pilkonis et al., 2011). The QIDS is a 16-item scale is used to assess depressive signs and symptoms scored on a 0–3 severity scale, with higher scores meaning higher depressive symptoms and cutoff points representing levels of severity for clinical depression: none, mild, moderate, and severe/very severe. Factors evaluated include sleep, psychomotor disturbance, appetite/weight changes, depressed mood, and concentration. The QIDS questions are derived from the DSM criteria for depression and thus have relevance for clinical depression. The QIDS has demonstrated to be both reliable (a = 0.80 to 0.94; Rush et al., 2003) and strongly correlated to other validated measures such as the Beck Depression Inventory and the Hamilton Rating Scale for Depression (Trivedi et al., 2004). The PROMIS-DEP is comprised of 28 items scored on a 0–5 scale (with greater scores meaning greater display of the symptoms) and cut-off points representing levels of symptom severity. It has been proven to be both valid and reliable across different samples to assess depression symptoms (Choi et al., 2014).

### Cognitive Battery Tasks

Executive Function was measured with the *Penn Computerized Neurocognitive Battery* (*CNB)* (Gur et al., 2010) which is composed of various tasks evaluating neurocognition across several domains. All the tasks were developed and validated by the University of Pennsylvania. The tasks took around an hour to complete in total. See below for a brief summary of the tasks.

#### The Penn Emotion Recognition Task (ERT)

The ERT is a measure of emotion recognition. Participants were shown different faces and asked to identify which emotion the person is feeling based on their facial expressions (happy, sad, anger, fearful or no emotion).

#### N-Back Test (n-Back)

This was used to measure attention and working-memory, utilizing three different trial types: the 0-back, in which participants respond whenever the letter X appears on the screen; the 1-back, in which participants respond whenever the letter on the screen is the same as the previous letter; and, the 2-back, in which participants must press the spacebar whenever the letter on the screen is the same as the letter before the previous letter.

#### The Penn Word Memory Test (WMT)

Participants are shown 20 words that they will be asked to identify later. During recall, participants are shown a series, one at a time, of 40 words. Participants decide whether they have seen the word using a 4-point scale: “definitely yes”, “probably yes”, “probably no” and “definitely no”.

#### The Penn Face Memory Test (FMT)

Participants are shown 20 faces that they will be asked to identify later. During recall, participants are shown a series, one at a time, of 40 faces. Participants decide whether they have seen the face using a 4-point scale: “definitely yes”, “probably yes”, “probably no” and “definitely no”.

#### Penn Conditional Exclusion Task (CET)

The CET is a measure of abstraction and mental flexibility. There are three principles/criteria for choosing an object: line thickness, shape, and size. The participant is not told what the ruling principle is and must derive the correct principle by clicking on an object and receiving feedback as to whether this is the correct response.

#### Short Penn Line Orientation Test (sPLOT)

The sPLOT measures complex cognition by probing visuospatial abilities. Participants were shown two lines (one adjustable and one fixed) in different orientations and were asked to rotate the adjustable line to make it parallel to the fixed line.

### Analysis

To quantify differences in mental health symptom burden and impulsivity by group (CSA-Y and CSA-N), SPSS software was used. Though the significance criterion was set to α = 0.05, we chose to present results using estimation methods, which avoids the issues with “null hypothesis significance testing” but also underscores the importance of effect sizes and confidence intervals (Williams et al., 2023). To assess whether there were group differences in demographic, health, and neurocognitive variables, we used two-sample *t* tests with percentile bootstrapping (1,000 samples) and 95% confidence intervals (CI) for variables that were continuous (e.g., age, depressions, etc.) or X^2^ tests for variables that were nominal (e.g., sex, education, drug use, etc.). If a variable (e.g., depression scores) differed between groups, this was controlled for in a separate analysis of putative differential cognitive performance using Quade non-parametric analysis of covariance (ANCOVA) in SPSS. In addition, we applied false discovery rate (FDR) correction to each set of analyses (e.g., demographics, correct responses, reaction time). In SPSS, Pearson correlations were run for normally distributed data, and Spearman correlations were run for non-normally distributed data. Distribution normality was tested with Shapiro-Wilk. Finally, to explore the relationships between significant depression variables, CSA, and significant neurocognitive measures, we used linear regression in SPSS. The first linear regression model tested for main effects, and the second regression model included an interaction term to test for moderating effects. In the model with interaction terms, it was necessary to mean center variables to reduce multicollinearity.

## Results

### Participants and Survey Results

Thirty-three individuals aged 31 to 60 years old (*M* = 45.56; *SD* = 8.67) participated in this study. There were 28 women and 5 men, with 15 identifying as white (2 identifying as Hispanic/Latinx), 15 as African American or Black, and 2 as mixed race (e.g. a combination of white, African-American or Black, and American Indian or Alaskan Native). One person chose not to provide their racial or ethnic background. Thirty participants self-described as having addiction and 26 self-reported having some type of mental health disorder, with 29 reporting using opioids (heroin *n* = 18, pain pills *n* = 17, and fentanyl *n* = 15) in the past year. Fifteen of the participants reported additional use of stimulants (e.g., cocaine, methamphetamine, prescription misuse). Twenty individuals reported having CSA. Individuals with a history of CSA (CSA-Y, *n* = 20) had significantly more self-reported mental health diagnoses than those without CSA (CSA-N, *n* = 13) (x2 = 7.98, *p* < 0.01) but did not differ on drug use measures. There were no other significant differences in demographic variables.

Depression results across our sample are as follows: a clinical assessment of depression, QIDS (M = 16.21, SD = 7.55), and a more symptomatic assessment of depression, PROMIS-DEP (M = 51.06, SD = 24.47). The CSA-Y group scored significantly higher on QIDS (M = 18.65) compared to those without (M = 11.77; t = −3.09, p-value = 0.004). These differences were not seen, however, when assessing depression using the PROMIS-DEP (CSA-N, M = 47.23, CSA-Y M = 53.55; t=−0.80, *p* = 0.43).

### Neurocognition Results

**Fig. 1:**
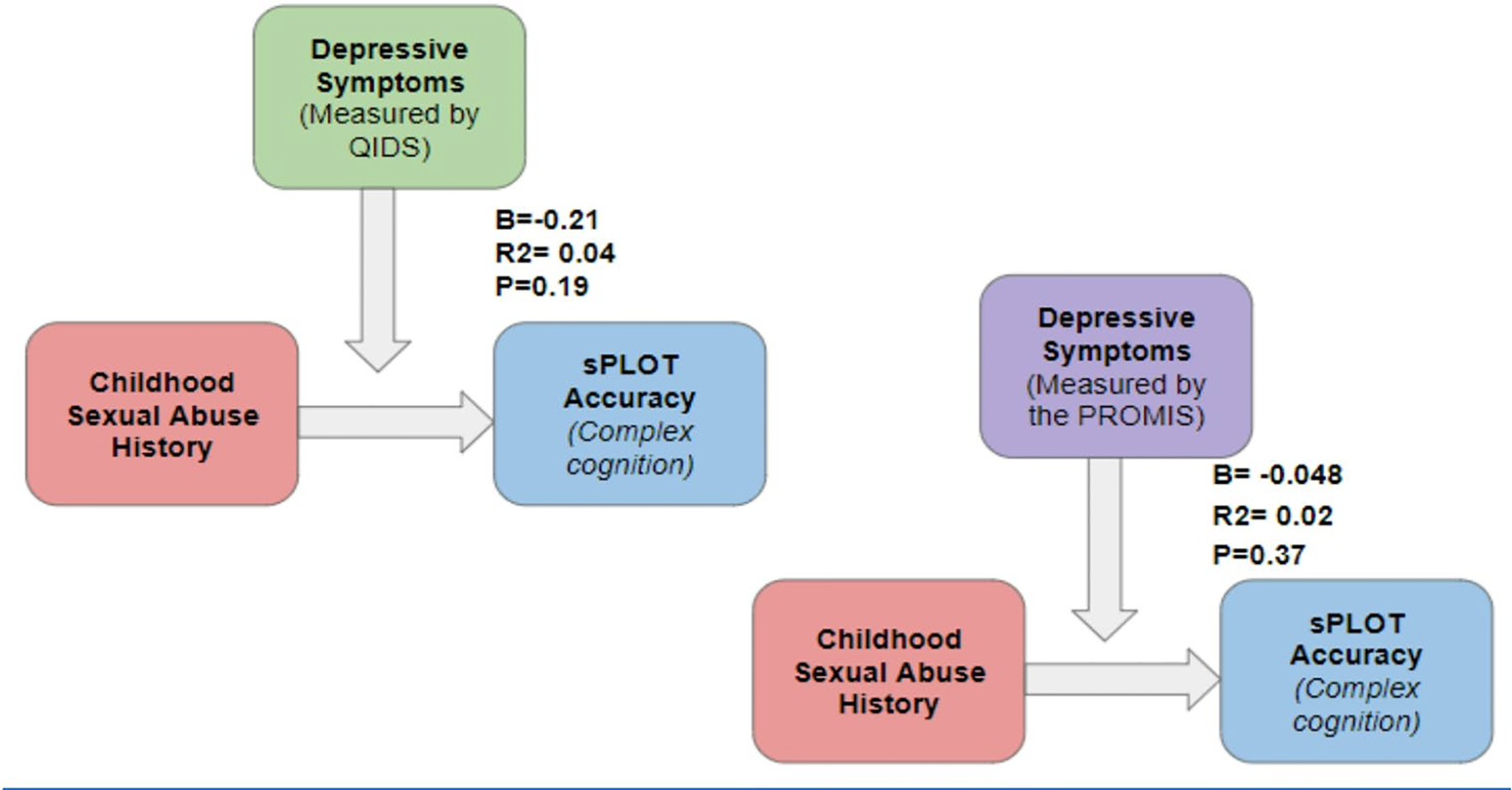
Moderation results comparing QIDS to PROMIS-DEP

There was a significant difference between CSA-Y vs. CSA-N for correct responses on the sPLOT (t = 2.34, *p* = 0.03, MD = 2.46 [0.5, 4.36], *g* = 0.86), with CSA-Y having fewer correct responses; however these results did not survive FDR correction. There were no other differences for other neurocognitive measures that reached the statistical threshold of *p* < 0.05 (See Table 1). The relationship between CSA and sPLOT, when controlling for self-reported history of a mental health diagnosis, remained significant (F(1,30) = 5.3, *p* = 0.03). When controlling for depression symptoms, the CSA / sPLOT relationship became insignificant when using the participants’ QIDS score (F(1,30) = 1.41, *p* = 0.25), however, it remained significant when using the participants’ PROMIS-DEP score (F(1,31) = 5.45, *p* = 0.026) (See Fig. 1).

## Exploratory Results: Potential Moderating Effect of CSA on the Relationship between Depressive Symptomatology and sPLOT Performance

**Fig. 2:**
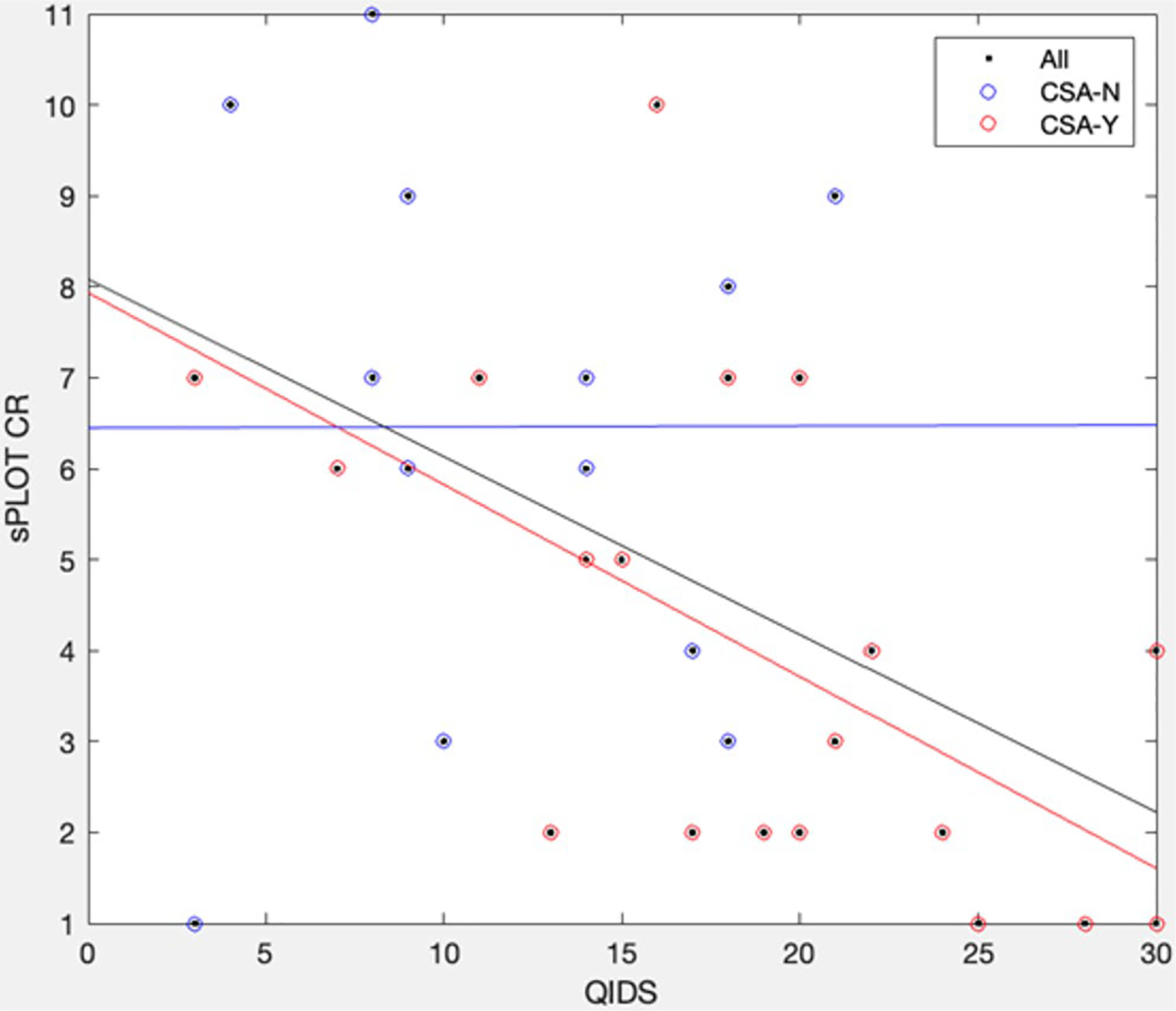
Correlation of QIDS and sPLOT CR for all (black, *R* = −0.46) participants and for CSA-Y (red, *R* = −0.62) and CSA-N (blue, *R* = −0.08) groups. Correlations between groups did not differ significantly (z = 1.59, *p* > 0.1)

To test the relationship between depression symptoms (QIDS), CSA, and sPLOT performance, exploratory analyses were conducted. First, a significant inverse correlation was found between QIDS and sPLOT performance across all participants (*R* = −0.46, *p* = 0.007; Fig. 2). When analyzing correlations between CSA groups, only CSA-Y showed a significant relationship (*R* = −0.62, *p* = 0.004), but CSA-N did not (Fig. 2a). Despite these differences, the between-group correlations did not significantly differ (z = 1.59, *p* > 0.10).

Next, we conducted a linear regression model with CSA and QIDS as independent variables and sPLOT performance as the dependent variable. The overall model was significant (R^2^ = 0.30, adjusted R^2^ = 0.25, F(2,30) = 6.35, *p* = 0.005). Within the model, QIDS demonstrated a significant negative relationship with sPLOT (B = −0.22, *p* = 0.03), while CSA was not a significant predictor (B = −1.44, *p* = 0.17).

Finally, to test whether CSA moderates the relationship between QIDS and sPLOT, an interaction term (CSA × QIDS) was added to the model. The revised model remained significant overall (R^2^ = 0.32, adjusted R^2^ = 0.25, F(3,29) = 4.53, *p* = 0.01). However, none of the individual predictors, including CSA (B = −1.84, *p* = 0.11), QIDS (B = −0.18, *p* = 0.08), or the interaction term (B = −0.22, *p* = 0.34), reached statistical significance. These results suggest that CSA does not significantly moderate the relationship between QIDS and sPLOT performance.

## Discussion

The present pilot study investigated the relationship between CSA history and its potential links with depressive symptoms and executive functioning in persons recovering from OUD. The findings provide valuable pilot data into the associations between CSA, depression, and neurocognition for those in recovery from OUD. These relationships should be further explored in larger samples to determine how a history of CSA might impact cognition and behavior for individuals in recovery.

Consistent with previous research (Mullen et al., 1993; Wilson, 2010; Zlotnick et al., 2001), individuals with a history of CSA reported significantly higher levels of clinical depression (via QIDS) compared to those without a CSA history, but levels of depression symptoms (via PROMIS-DEP) did not differ. This result aligns with the existing literature, which has demonstrated that CSA is a risk factor for various mental health concerns, particularly clinical depression. The pilot also examined executive functioning among participants with and without a CSA history. The results suggested those with CSA history had significantly fewer correct responses on the Short Penn Line Orientation Test (sPLOT) compared to those without CSA history; however this result did not survive FDR correction. This finding may suggest that a history of CSA might be associated with specific deficits in complex cognition among individuals in recovery from OUD, specifically relating to spatial orientation abilities; however, more data is needed to confirm this finding.

When controlling for the impact of depression, we observed differing results depending on the measure used. Using the Quick Inventory of Depressive Symptomatology (QIDS), the relationship between CSA and sPLOT became statistically insignificant. However, this was not the case when using the PROMIS Depression scale (PROMIS-DEP). This discrepancy might be attributed to variations in the sensitivity and specificity of different depression measures. The PROMIS-DEP (Pilkonis et al., 2011) focuses more on the mental and emotional aspects of depression (e.g., hopelessness, sadness, guilt), whereas the QIDS (Rush et al., 2003) aligns more closely with clinical criteria for depression as outlined in the DSM-IV, focusing on physical and cognitive manifestations (e.g., concentration difficulties, sleep disturbances, energy challenges). The inclusion of two depression screening tools allowed us to capture a more comprehensive picture of depressive symptoms, potentially uncovering nuances in how depression manifests in individuals with CSA and OUD histories.

Our exploratory analysis examined the relationships between sPLOT performance, history of CSA, and depressive symptoms. While QIDS scores and sPLOT performance were negatively correlated overall, only the CSA-Y group (but not CSA-N) showed a significant inverse relationship, suggesting a potential moderating effect of CSA. However, when tested formally in a regression model, the interaction term (CSA × QIDS) did not reach significance, indicating insufficient evidence to conclude moderation. The regression model testing main effects of QIDS and CSA on sPLOT performance was significant overall, with QIDS emerging as a significant predictor, while CSA was not. These findings suggest that individuals with higher levels of depression, particularly those with a history of CSA, may exhibit poorer performance on a complex cognition task. However, the lack of significant interaction or CSA-specific effects underscores the need for additional data to clarify these relationships.

The clinical significance of the sPLOT results lies in their potential to better understand the impact of CSA and associated consequences on daily functioning. Spatial orientation abilities are crucial for activities such as navigation, driving, and certain job-related tasks (e.g., Morrissey et al., 2014). Deficits in this area could potentially impact an individual’s ability to function effectively in various environments, which could, in turn, affect their recovery process. However, it’s important to note that many individuals with CSA histories demonstrate remarkable resilience and adaptability, often developing compensatory strategies to overcome such challenges. The term “complex cognition” refers to higher-order cognitive processes that integrate multiple cognitive domains. CSA might affect these processes more than other neurocognitive measures due to its potential impact on brain development and functioning, particularly in areas related to executive function and spatial processing.

These findings suggest the importance of considering the interplay between mental health factors and cognitive outcomes when studying the impact of childhood trauma on neurocognitive functioning. They also highlight the complexity of the relationships between trauma history, depressive symptoms, and cognitive performance, underscoring the need for comprehensive assessment in clinical practice. Moreover, these results point to the possibility that individuals with a history of CSA might be more vulnerable to the cognitive impacts of depression, particularly in domains like spatial orientation and visuospatial processing. The discrepancy between QIDS and PROMIS-DEP results, coupled with the association between QIDS and sPLOT performance, suggests that CSA might have a more direct impact on neurocognitive functioning than initially hypothesized, potentially independent of depressive symptoms. Previous studies suggest CSA is associated with changes in the posterior cortex (e.g., visual cortex) (Tomodo et al., 2009), and this brain region underlies sPLOT performance. Thus, CSA might affect processing of sensory information as well as the ability to process multiple inputs (e.g., visuospatial), leading to potential system “overload”, though more studies are needed.

It’s important to note that while our study focused on depression, posttraumatic stress disorder (PTSD) is also strongly associated with CSA and has known neurocognitive impacts (e.g., Boumpa et al., 2024). Our decision to focus on depression was based on its high prevalence in OUD populations and its established relationship with cognitive functioning. However, future studies should consider including PTSD measures to provide a more comprehensive understanding of the psychological impacts of CSA in this population.

### Implications

The findings of this brief report carry several important implications for clinical practice, research, and interventions aimed at supporting individuals in recovery from OUD with a history of CSA. These findings underscore the necessity of trauma-informed care for individuals in OUD recovery, acknowledging the potential impact of CSA on mental health and cognitive functioning. Trauma-informed interventions should be integrated into substance use treatment programs to address the unique needs and challenges faced by individuals with a history of CSA. Specific approaches could include utilizing Eye Movement Desensitization and Reprocessing (EMDR) therapy (Martínez-Fernández, 2024) which has been found to not only improve trauma symptomology, but also to significantly reduced the desire to consume drugs, incorporating mindfulness-based interventions like Mindfulness-Based Relapse Prevention (MBRP) (Bowen et al., 2021), and providing psychoeducation about the links between trauma, mental health, and substance use. In addition, the results suggest that interventions targeting neurocognitive difficulties may also be useful for those in recovery from OUD. Proof of concept studies on cognitive training for substance use disorders have shown promise and may improve underlying neural mechanisms (Verdejo-Garcia, 2016). Specific cognitive interventions could include computerized cognitive remediation programs (Rezapour et al., 2017), Goal Management Training (Alfonso et al., 2011), and attention bias modification training which has shown reductions in attentional bias for drug-related stimuli, temptations to use, and number of lapses, and increases on readiness to change in those using opiates (Ziaee et al., 2016). These interventions should be tailored to address both substance-related and trauma-related cognitive challenges, potentially improving treatment outcomes for individuals with comorbid CSA history and OUD.

While many programs already include CSA screening during OUD evaluation, the critical next step is effectively using this information to support individuals. Clinicians should use CSA history to inform personalized treatment plans that address both substance use and trauma-related issues. This could involve integrating trauma-specific therapies (e.g., Trauma-Focused Cognitive Behavioral Therapy) alongside substance use treatment, and being mindful of potential triggers or challenges that may arise during recovery.

Moreover, clinicians should be aware of the potential cognitive impacts of CSA, particularly in areas of complex cognition. Treatment approaches may need to be adapted to accommodate these cognitive challenges. For instance, providing written materials to supplement verbal instructions, using visual aids, or breaking down complex tasks into smaller, manageable steps could be beneficial. These adaptations could significantly improve the effectiveness of treatment and support individuals in their recovery process.

It’s crucial to recognize the strengths and resilience of this population. Many individuals with histories of CSA and OUD demonstrate remarkable resilience in their recovery journey. Treatment approaches should aim to build upon these strengths, fostering resilience and empowering individuals in their recovery process. This could involve identifying and reinforcing positive coping strategies, celebrating small victories, and promoting peer support networks.

The association between CSA and higher levels of depression underscores the need for treatment providers to be vigilant in assessing and addressing mental health issues among individuals in OUD recovery. While our study focused on depression, clinicians should be aware that other mental health issues, including PTSD, may also be prevalent in this population. A comprehensive mental health assessment, including both depression and PTSD symptoms, can provide a more complete picture of an individual’s psychological state and inform more targeted interventions.

Regarding the potential direct impact of CSA on neurocognitive performance, it’s important to note that while our results suggest a relationship between CSA and cognitive functioning, the role of depression cannot be discounted. The complex interplay between trauma, mental health, and cognitive functioning underscores the need for integrated treatment approaches that address all these aspects simultaneously.

The differences in the instruments used for depressive symptom assessment may not imply one is superior to the other, but rather that each instrument is tailored to emphasize distinct aspects of depression. Researchers and clinicians should carefully consider the specific research question and the aspects of depression they aim to investigate when selecting appropriate depressive symptom assessment instruments in the future. Further research and replication studies are necessary to corroborate the observed differential effects.

### Limitations

This study was a pilot study and thus was limited by small sample sizes and recruitment through organizations supporting those in recovery from OUD. This may have biased our results, as those who are actively engaging in services for recovery may be fundamentally different from those who are not currently engaged in services. In addition, our study was limited by behavioral data only, and future research should explore reactivity at the level of the brain to help make greater sense of the relationships found. It is possible differences in neurocognition could be due to other factors (e.g., medical, overdose-related); however, this pilot study did not capture those events. The sample was predominantly women-identifying, which may limit generalizability to male and other gendered populations.

An additional limitation of our study was the use of a single dichotomous variable for CSA. This approach doesn’t account for the potential differential impacts of penetrative versus non-penetrative CSA, the age at which the abuse occurred, or the chronicity/multiple exposures to abuse. These factors could have varying effects on cognitive and psychological outcomes. Future studies should consider using more nuanced measures of CSA to capture these important distinctions.

While our study focused on depression, posttraumatic stress disorder (PTSD) is also strongly associated with CSA and has known neurocognitive impacts. Our decision to focus on depression was based on its high prevalence in OUD populations and its established relationship with cognitive functioning. However, future studies should consider including PTSD measures to provide a more comprehensive understanding of the psychological impacts of CSA in this population.

While preliminary results highlight potential links between CSA, depression, and sPLOT performance, the small sample size limits definitive conclusions. Future research with larger samples is needed to further investigate these trends and better understand the interplay between CSA, depression, and cognitive performance. The complex interplay between trauma, mental health, and cognitive functioning underscores the need for integrated treatment approaches that address all these aspects simultaneously.

Neurocognitive performance could be affected by severity/chronicity of drug use, or substance-related adverse events such as hypoxia from overdose or traumatic brain injury through violent assaults or accidents while intoxicated. Future studies should aim to control for these factors. Despite these limitations, this study offers a foundation for future research and intervention development to support those in recovery from OUD.

## Conclusion

This pilot study provides valuable insights into the complex interplay between CSA, depression, and cognitive functioning in individuals in recovery from OUD. These results emphasize the importance of considering the complex interrelationships between childhood trauma, mental health, and cognitive outcomes for those recovering from OUD. By recognizing and addressing the intersecting impacts of childhood sexual abuse and opioid use disorder, treatment professionals can facilitate better outcomes and promote a more personalized, healing-centered approach for individuals on their path to recovery.

## Figures and Tables

**Table 1:** Computerized neurocognitive Battery - Performance results (CSA-N > CSA-Y)

Task	*Overall (n = 33)*	*CSA-N (n = 13)*	*CSA-Y (n = 20)*	Mean Difference & 95% Confidence Intervals	T & *P* Values	Effect Size
***Emotional Recognition***						
*Correct Responses*	*29.3*	*30.1*	*28.8*	*1.28 [−1.68, 4.2]*	*t = 0.85 (p = 0.40)*	*0.3*
*Reaction Time (ms)*	*2537*	*2462*	*2587*	*−125 [−498, 236]*	*t = 0.66 (p = 0.52)*	*−0.23*
***Word Memory Test***						
*Correct Responses* *	*30.4*	*30.5*	*30.4*	*0.19 [−2.68, 3.17]*	*t = 0.13 (p = 0.90)*	*0.04*
*Reaction Time (ms)*	*1961*	*1930*	*1992*	*−62 [−396, 246]*	*t = 0.37 (p = 0.99)*	*0.12*
***Conditional Exclusion Test***						
*Accuracy*	*1.11*	*1.08*	*1.13*	*−0.04 [−0.52, 0.42]*	*t = 1.09 (p = 0.28)*	*0.07*
*Reaction Time (ms)* *	*3110*	*3354*	*2866*	*488 [−345*, *1286]*	*t = 1.19 (p = 0.25)*	*0.47*
***Face Memory Test***						
*Correct Responses*	*27.6*	*27.2*	*28*	*−0.77 [−4.45, 2.71]*	*t = 0.41 (p = 0.68)*	*0.14*
*Reaction Time (ms)*	*1845*	*1816*	*1874*	*−58 [−322*, *204]*	*t = 0.43 (p = 0.67)*	*0.15*
***N-Back Task***						
*True Positives* *	*15.7*	*17*	*14.3*	*2.74 [0.12, 5.54]*	*t = 2.0 (p = 0.06)*	*0.61*
*Reaction Time (ms)*	*660*	*621*	*699*	*−78 [−194, 34]*	*t = 1.3 (p = 0.21)*	*0.45*
***Spatial Line Orientation Task***						
*Correct Responses*	*5.25*	*6.5*	*4*	*2.46 [0.50, 4.36]*	*t = 2.34 (p = 0.03)*	*0.86*
*Reaction Time (ms)*	*16,293*	*14,434*	*18,151*	*−3717 [−10943, 2970]*	*t = 0.66 (p = 0.52)*	*0.33*

* Levene’s test for equality = < 0.05

## Data Availability

Data can be made available upon request.
